# Phase 2 Trial of PD‐1 Inhibitor Sintilimab in Recurrent/Progressive Meningioma

**DOI:** 10.1111/cns.70659

**Published:** 2025-12-08

**Authors:** Yali Wang, Can Wang, Shuo Yin, Chunna Yu, Xiaojie Li, Xun Kang, Shoubo Yang, Wenting Xie, Yi Lin, Zhen Wu, Wenbin Li, Feng Chen

**Affiliations:** ^1^ Department of Neuro‐Oncology, Cancer Center Beijing Tiantan Hospital, Capital Medical University Beijing China; ^2^ Hepato‐Pancreato‐Biliary Center, School of Clinical Medicine, Beijing Tsinghua Changgung Hospital Tsinghua University Beijing China; ^3^ Beijing Luhe Hospital Capital Medical University Beijing China; ^4^ Department of Neurosurgery Beijing Tiantan Hospital, Capital Medical University Beijing China

**Keywords:** meningioma, PD‐1 inhibitor, phase 2 trial, progressive, recurrent

## Abstract

**Background:**

Systemic therapeutic options for meningiomas remain limited. Emerging evidence indicates meningiomas harbor an immunosuppressive microenvironment and programmed cell death ligand 1 (PD‐L1) expression is significantly upregulated in both tumor cells and tumor‐infiltrating immune cells. Here we conducted a single‐arm, single‐center, open‐label, phase 2 clinical trial (NCT 04728568) evaluating the programmed cell death receptor‐1 (PD‐1) inhibitor sintilimab in patients with recurrent/progressive meningiomas following standard surgery and/or radiotherapy.

**Methods:**

Forty patients (9 grade 1, 18 grade 2, and 13 grade 3) received intravenous sintilimab (200 mg every 3 weeks). According to Response Assessment in Neuro‐Oncology for meningioma (RANO‐meningioma) criteria, the 6‐month progression‐free survival rate (PFS‐6) was used as the primary endpoint. Secondary endpoints included the 12‐month progression‐free survival rate (PFS‐12), PFS, overall survival (OS), and safety. Peripheral lymphocyte subpopulations, tumor‐infiltrating lymphocyte (TIL) densities, and tumor mutational burden (TMB) were evaluated as immunocorrelated biomarkers.

**Results:**

Patients with grade 1 exhibited a PFS‐6 of 67.0%, a PFS‐12 of 56.0%, and the median PFS was 14 months (95% CI: 0, 31.5). Grade 2/3 patients showed a PFS‐6 of 42.0%, a PFS‐12 of 19.0%, and the median PFS was 5.0 months (95% CI: 3.46, 6.54). The median OS was 27.0 months (95% CI: 17.26, 36.73) in grade 2/3 patients. The best outcome among all patients was stable disease (SD). Sintilimab was well tolerated without severe adverse events. A patient with a high TMB (13.14 muts/Mb) had a pseudoprogression with sintilimab and maintained stable disease among subsequent treatments. Among 3 patients with matched pre‐/post‐treatment tumor samples, 2 showed increased PD‐1+ T cell expression after sintilimab.

**Conclusion:**

Sintilimab failed to improve PFS‐6 in both grade 1 and grade 2/3 recurrent/progressive meningiomas in this single‐arm, single‐center, and small‐sample trial. When evaluating PD‐1 inhibitor treatment for recurrent/progressive meningioma patients, who generally have a longer expected survival and high TMB, the use of the Immunotherapy Response Assessment in Neuro‐Oncology (iRANO) criteria may be more appropriate to avoid overlooking potential clinical benefits.

## Graphical abstract

First trial evaluating PD‐1 inhibitors in WHO grade 1 meningioma (*n* = 9). Largest grade 2/3 meningioma cohort treated with PD‐1 inhibitors (*n* = 31). PFS‐6 was 42.0% in grade 2/3 cases and 67.0% in grade 1 cases. No new safety signals observed.

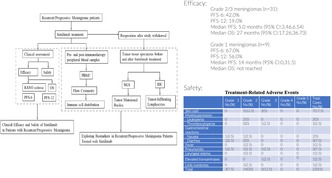

**Abbreviations**
IHC: immunohistochemistry
NGS: next‐generation sequencing
OS: overall survival
PBMC: peripheral blood mononuclear cell
PFS: progression‐free survival
TMB: tumor mutation burden

## 1 Introduction

Meningiomas represent the most common primary intracranial neoplasms, accounting for approximately one‐third of all primary central nervous system tumors [1, 2]. According to the World Health Organization (WHO), the majority of meningiomas (80%–95%) are classified into low grade (grade 1), while a smaller proportion are classified as high grade (grade 2: 5%–15%, grade 3: 1%–3%) [3, 4, 5]. These higher‐grade meningiomas demonstrate aggressive clinical behavior, with recurrence rates reaching 30%–40% for grade 2 and up to 80% for grade 3 tumors. The prognosis remains poor, with 5‐year overall survival rates of only 60% and 30% for grade 2 and 3 meningiomas, respectively [6, 7, 8, 9]. Despite multimodal treatment approaches including multiple surgical resections and radiotherapy, disease progression remains a significant challenge for many patients. Currently, systemic treatment options for meningiomas are limited, highlighting the urgent need for developing more effective therapeutic strategies.

In recent years, immune checkpoint inhibitors (ICIs) have achieved remarkable results in treating various solid tumors. Specifically, monoclonal antibodies targeting programmed cell death receptor‐1 (PD‐1) and programmed cell death ligand 1 (PD‐L1) have shown significant clinical benefits in malignancies such as lung cancer and melanoma [10]. However, current clinical guidelines do not include PD‐(L)1 inhibitors as recommended treatment options for meningiomas, highlighting an important gap in therapeutic strategies for these intracranial tumors.

Growing evidence suggests that meningiomas possess an immunosuppressive tumor microenvironment. Notably, PD‐L1 expression is significantly upregulated in both tumor cells and tumor‐infiltrating immune cells, with expression levels correlating positively with histological grade [10, 11, 12, 13]. This biological rationale is supported by promising responses to PD‐1 inhibitors observed in case reports of recurrent meningiomas [14, 15]. These findings provide a strong mechanistic basis for investigating PD‐1 blockade in recurrent meningiomas, particularly in high‐grade cases. However, large‐scale clinical studies evaluating PD‐1 inhibitors in this population remain lacking, and both therapeutic efficacy and underlying mechanisms require systematic investigation.

## 2 Patients and Methods

This is a single‐arm, single‐center, open‐label, phase 2 study conducted in Beijing Tiantan Hospital (NCT 04728568). The complete study protocol is available in the Appendix S1. Patients with histologically confirmed meningioma who have received prior standard therapies (including surgery and/or radiotherapy) and are deemed by the investigator to be ineligible for further surgical intervention or radiotherapy at the current stage may be enrolled if they meet the following criteria for recurrence or progression: (a) Recurrent disease: Clear tumor recurrence or progression after surgical resection. (b) Progressive disease: Documented radiographic evidence of disease progression following the last medical intervention (surgery, radiotherapy, or drug therapy) in patients who have undergone prior surgery, or surgery combined with radiotherapy. Participants with residual lesions after surgery may be considered for inclusion if preoperative imaging shows clear progression compared to prior imaging, and the residual lesions meet the measurable lesion defined by the Response Assessment in Neuro‐Oncology‐meningioma (RANO‐meningioma) criteria [16] (both the longest diameter and its perpendicular greatest short diameter ≥ 10 mm).

Inclusion criteria were as follows: aged ≥ 18 years old; had a Karnofsky Performance Status (KPS) of at least 60; with adequate organ function. Exclusion criteria comprised: corticosteroid dependence (> 5 mg/day dexamethasone or equivalent prednisone dose); PD‐(L)1 inhibitor exposure within 6‐month washout period; radiographic progression within 12 weeks postradiation or < 4‐week washout from prior systemic agents (whichever shorter); primary spinal meningiomas; active autoimmune disorders requiring systemic immunosuppression within 3 months; absolute PD‐1 inhibitor contraindications.

This study was conducted in strict accordance with the ethical principles outlined in the Declaration of Helsinki and Good Clinical Practice guidelines. The study protocol received ethical approval from the Institutional Review Board of Beijing Tiantan Hospital, Capital Medical University, and written informed consent was obtained from all participating patients.

### 2.1 Treatment Plan

In this prospective study, patients received intravenous sintilimab (PD‐1 inhibitor, Innovent Biologics/Eli Lilly, China), at a fixed dose of 200 mg administered every 3 weeks (Q3W). Treatment continuation was subject to three discontinuation criteria: (1) radiographic evidence of disease progression as per RANO‐meningioma criteria, (2) development of intolerable treatment‐related adverse events, or (3) voluntary withdrawal of consent. For comprehensive immunological monitoring, peripheral blood samples were systematically collected at key timepoints: baseline (pretreatment), 6 weeks (after 2 cycles), 9 weeks (after 3 cycles), and at each subsequent treatment session, including during any period of therapy interruption. The collected specimens underwent multiparameter analysis including: (1) complete hematological profiling, (2) comprehensive metabolic panel assessment, and (3) flow cytometric quantification of lymphocyte subsets (CD3 + CD4+ T cells, CD3 + CD8+ T cells and NK cells).

Contrast‐enhanced MRI scans were conducted at baseline and every 6–9 weeks throughout the treatment period, with additional scans conducted when clinical evaluation suggested significant tumor regression or progression. All radiographic assessments were interpreted according to the RANO‐meningioma [16, 17]. Six‐month progression‐free survival rate (PFS‐6) was used as the primary endpoint according to a meta‐analysis conducted in 2014 by the RANO group [18]. The review reported that for grade 1 meningioma, the weighted average PFS‐6 was 29%, while for grade 2/3 the weighted average PFS‐6 was 26% (95% CI: 19.3%–32.7%). A threshold of PFS‐6 exceeding 50% and 35% was defined as indicative of further clinical study consideration for patients with grade 1 and grade 2/3 meningiomas, respectively. Secondary endpoints included 12‐month progression‐free survival rate (PFS‐12), PFS, overall survival (OS), and safety. PFS was defined as the duration from the initiation of PD‐1 inhibitor to disease progression, while OS was defined as the time from the start of PD‐1 inhibitor to death. Toxicity was graded using Common Terminology Criteria for Adverse Events version 5.03.

### 2.2 Biomarker Analyses

Peripheral blood samples were collected in EDTA anticoagulant tubes both before treatment initiation and following 2 cycles of PD‐1 inhibitor therapy. We performed comprehensive lymphocyte subset analysis by flow cytometry to evaluate treatment‐induced changes in the peripheral immune landscape at our clinical research center.

In patients undergoing additional surgeries following consent withdrawal, tumor specimens were preserved as formalin‐fixed, paraffin‐embedded (FFPE) blocks. Pretreatment FFPE slides from these cases were subsequently analyzed through standardized immunohistochemical staining utilizing antibodies targeting CD3, CD4, CD8, CD68, and PD‐1 to assess comparative changes in tumor‐infiltrating lymphocyte (TIL) proportions before and after therapeutic intervention.

Tumor mutational burden (TMB) was quantified from clinical genomic reports based on next‐generation sequencing (NGS) data of surgically resected specimens obtained prior to immunotherapy. The analysis utilized the Illumina NGS platform with a hybrid‐capture panel covering the full exonic regions of approximately 21,000 genes, including 830 clinically significant cancer‐related genes.

### 2.3 Statistical Analysis

This study employed a modified single‐arm phase 2 design. For grade 2/3, based on the PFS‐6 threshold (26%) established by the RANO working group, we hypothesized that sintilimab could increase PFS‐6 to 51% (absolute improvement of 25%). The original design, with a one‐sided *α* of 0.10 (exact *α* = 0.089), required 25 patients to provide 90% statistical power, setting the success criterion as ≥ 10 cases (40%) achieving PFS‐6. During the enrollment period, we were able to identify and enroll a total of 31 eligible patients consecutively, and thus included all of them in the final analysis to maximize data utility. After actual enrollment of 31 patients, recalculation via binomial distribution indicated that maintaining a comparable *α* level necessitated adjustment of the success threshold to ≥ 12 cases (38.7%, exact *α* = 0.075). Observation of outcomes meeting this threshold would lead to rejection of the null hypothesis and support further investigation of this therapeutic approach. For the grade 1 cohort, due to the scarcity of recurrent/progressive cases, a formal sample size calculation was not pre‐specified. Instead, we adopted a strategy to enroll all eligible patients available during the study period, which resulted in a final cohort of nine patients for exploratory analysis.

Normality‐confirmed paired continuous variables were analyzed using paired Student's *t*‐tests (Shapiro–Wilk verification, two‐tailed *α* = 0.05). Time‐to‐event outcomes were visualized through Kaplan–Meier survival estimates with log‐rank comparative analysis. Statistical computations were executed in SPSS 26.0, R‐4.5.1 and GraphPad Prism 9.2.0 platforms.

## 3 Results

### 3.1 Patients and Treatment

**FIGURE 1:**
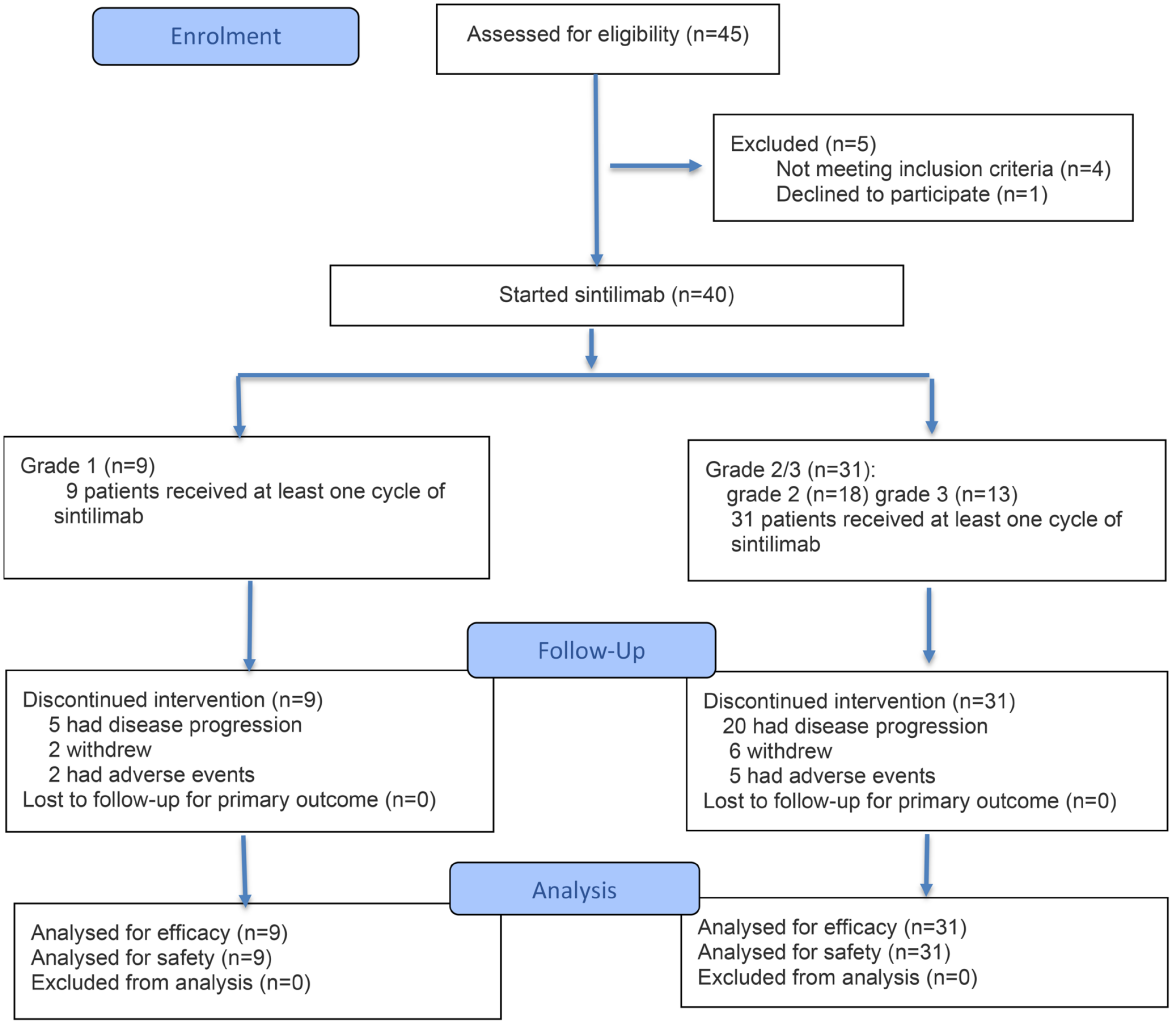
Flow diagram of the trial.

From August 2020 to January 2025, a total of 45 patients with recurrent/progressive meningiomas were assessed for eligibility and 5 of them were excluded because of not meeting inclusion criteria (*n* = 4) or declined to participate (*n* = 1). Ultimately, 40 patients were enrolled in this study, undergoing a cumulative 197 cycles of PD‐1 inhibitor monotherapy. The screening and enrollment process is summarized in the flow diagram (Figure 1). The clinical characteristics of the cases are shown in Table 1. The cohort included 9 (22.5%) grade 1, 18 (45.0%) grade 2, and 13 (32.5%) grade 3 tumors. The median age of the patients was 47.5 years (ranging from 18 to 83 years), with a majority of 23 (57.5%) being female. At the initiation of treatment, the patients had a median KPS score of 80 (ranging from 60 to 100).

**TABLE 1:** Clinical characteristics of all patients.

Characteristic	No. of patients (%)
Age	
≥ 47.5	20 (50.0)
< 47.5	20 (50.0)
Gender	
Female	23 (57.5)
Male	17 (42.5)
Grade at enrollment	
1	9 (22.5)
2	18 (45.0)
3	13 (32.5)
KPS (%)	
≥ 80	26 (65.0)
60–80	14 (35.0)
Tumor multifocality	
Multifocal (%)	14 (35.0)
Unifocal tumor	26 (65.0)
Tumor location (%)	
Supratentorial	11 (27.5)
Skull base	25 (62.5)
Both	4 (10.0)
No. of prior PD (%)	
≥ 3	23 (57.5)
< 3	17 (42.5)
No. of prior resections (%)	
≥ 2	25 (62.5)
< 2	15 (37.5)
No. of prior radiation courses (%)	
0	13 (32.5)
1	17 (42.5)
≥ 2	10 (25.0)
Time from diagnosis to enrollment (months)	
≥ 60.2	19 (47.5)
< 60.2	21 (52.5)
Ki‐67	
20–80	7 (17.5)
10%–20%	11 (27.5)
10%	5 (12.5)
≤ 5%	6 (15.0)
Unknown	11 (27.5)

Abbreviations: KPS, Karnofsky Performance Status; PD, progressive disease.

Intracranial meningioma distribution showed skull base localization in 25 cases (62.5%), supratentorial involvement in 11 cases (27.5%), and combined locations in 4 cases (10.0%). The median number of previous progression instances was 3 (ranging from 1 to 8). The median number of prior surgeries was 2 (ranging from 1 to 7), the median number of radiotherapies was 1 (ranging from 0 to 7), and 3 patients (7.5%) had received prior systemic therapy. The specific chemotherapy regimen consisted of carboplatin (100 mg/m^2^/day on days 1–3) combined with etoposide (100 mg/m^2^/day on days 1–3), with a 28‐day treatment cycle. The three patients received 2, 3, and 4 cycles (patient 8, 33, and 34) of this regimen, respectively. Patient 34 with cerebral edema concurrently received bevacizumab (5 mg/kg per administration) in addition to the chemotherapy, also on a 28‐day cycle for 3 cycles. The median duration from initial diagnosis to enrollment was 60.2 (ranging from 4 to 240) months.

Ki‐67 indices were derived from immunohistochemical testing of the most recent surgical tumor tissue specimen in 29 patients, with a median of 20.0% (ranging from 0% to 60%). The median number of treatment cycles with PD‐1 inhibitors was 4 (ranging from 2 to 17).

**FIGURE 2:**
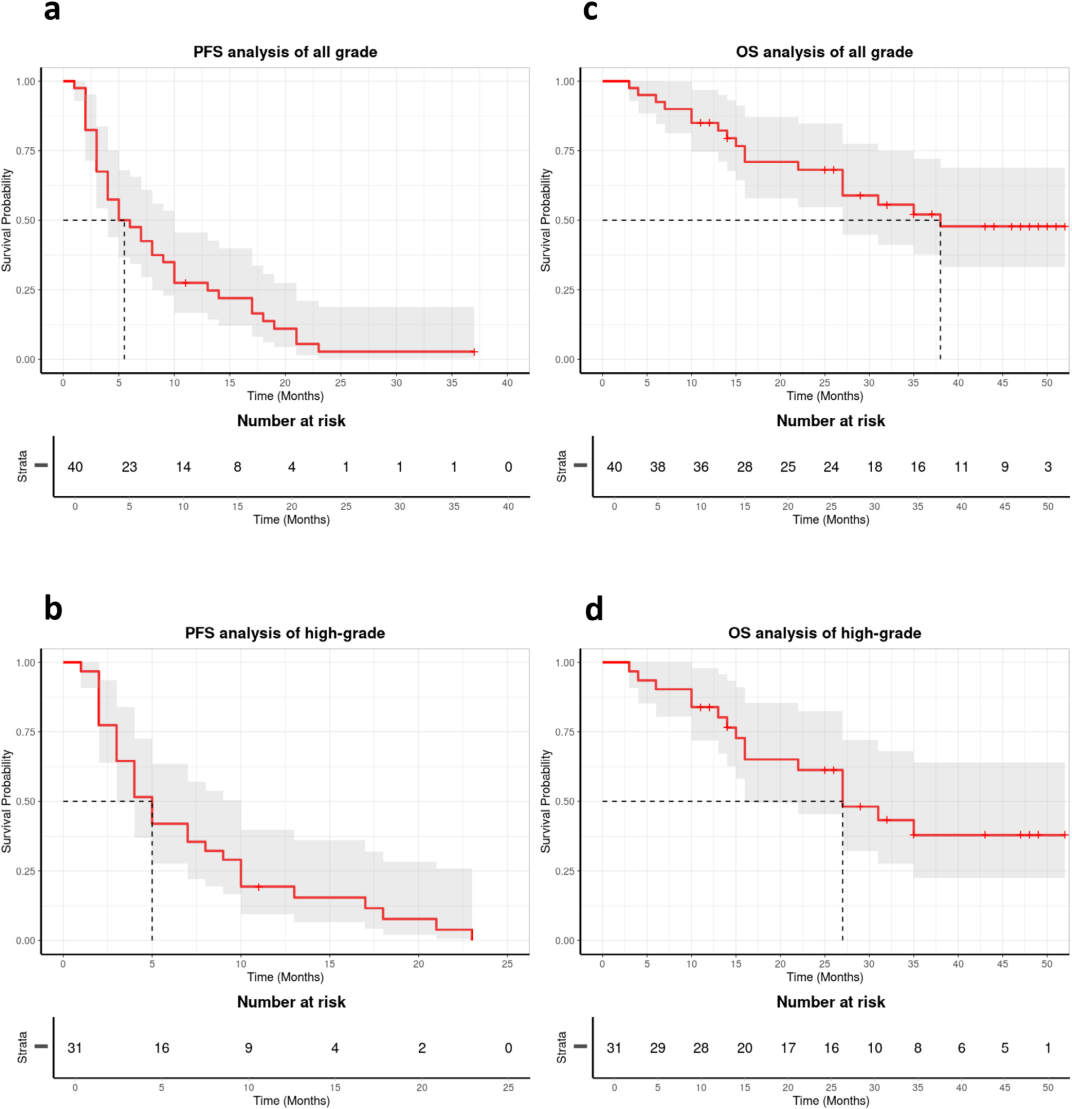
Kaplan–Meier curve. (a) PFS of all patients. (b) PFS of high‐grade patients. (c) OS of all patients. (d) OS of high‐grade patients.

**FIGURE 3:**
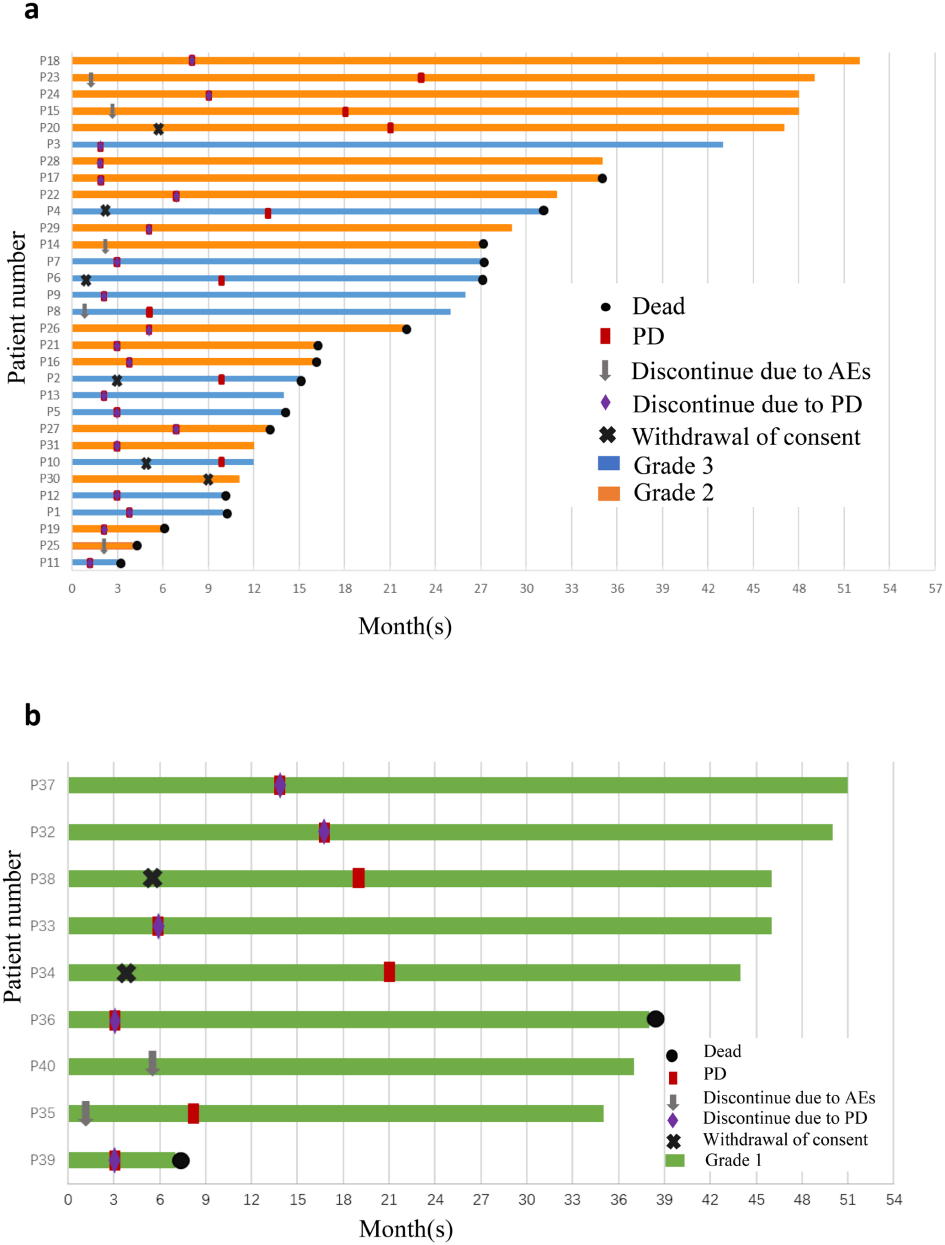
Treatment outcome event history by histopathologic grade (per patient). (a) High‐grade meningioma patients. (b) Low‐grade meningioma patients.

As of January 2025, 22 (55.0%) patients survived and 18 (45.0%) died. The median follow‐up was 38.0 (3.0–52.0) months. We performed survival analysis of PFS and OS of all grades (40 patients), grade 1 patients (9 patients), and grade 2/3 patients (31 patients), respectively. In patients with all grades, the cohort demonstrated a PFS‐6 of 47.0%, a PFS‐12 of 27.0%, and a median PFS of 5.0 months (95% CI: 1.90–8.10) (Figure 2a). Patients with grade 1 exhibited a PFS‐6 of 67.0%, a PFS‐12 of 56.0% and the median PFS was 14 months (95% CI: 0, 31.5). Grade 2/3 patients showed a PFS‐6 of 42.0%, a PFS‐12 of 19.0%, and the median PFS was 5.0 months (95% CI: 3.46, 6.54) (Figure 2b). The median OS was 38 months (95% CI: 29.01, 40.85) of all grades (Figure 2c) and 27 months (95% CI: 17.26, 36.73) in grade 2/3 patients (Figure 2d). The best outcome among all patients was stable disease (SD); no patient achieved partial response (PR) or complete response (CR). Twenty‐eight (70.0%) patients had SD and 12 (30.0%) patients had PD. Among the high‐grade group, 21 (67.7%) patients achieved SD and 10 (32.3%) patients had PD. Figure 3 displays patient‐level PFS and OS outcomes, stratified by initial WHO tumor grade (Figure 3a: grade 2/3 meningiomas; Figure 3b: grade 1 meningiomas). We further assessed potential prognostic factors in Table 1 for PFS and OS in all patients and grade 2/3 cases (age, gender, grade, KPS, tumor multifocality, tumor location, prior PD times, prior resection times, prior radiation courses, time from diagnosis to enrollment, and Ki‐67), but found no significant associations.

### 3.2 Safety

Treatment‐related adverse events (TRAEs) during and after treatment were recorded and followed up in 40 patients. Seventeen patients experienced 22 TRAEs of different degrees, with rash being the most common (7/40, 17.5%), followed by gastrointestinal reactions (5/40, 12.5%), myelosuppression (3/40, 7.5%), and the rest including fever, pneumonitis, laryngeal edema, and numbness of the limbs, most of which were relatively mild and did not exceed grade 2 (Table 2). Immune‐related adverse events (irAEs) are summarized separately in Table 3. Upon identification of a grade 3 adverse event, sintilimab treatment was discontinued and symptomatic treatment was initiated. For grade 2 adverse events, immune therapy was initially suspended and symptomatic management was implemented. If the adverse reaction resolved to grade ≤ 1 (mild) or completely returned to baseline levels, treatment was recommenced; otherwise, it was discontinued. Seven patients discontinued treatment due to grade 2 (*n* = 2) or grade 3 (*n* = 5) adverse reactions (rash, laryngeal edema, thrombocytopenia, pneumonitis, and elevated transaminases).

**TABLE 2:** Treatment‐related adverse events.

	Grade 1	Grade 2	Grade 3	Grade 4	Grade 5	Total cases
	No. (%)	No. (%)	No. (%)	No. (%)	No. (%)	No. (%)
Skin rash	0	5 (12.5)	2 (5)	0	0	7 (17.5)
Myelosuppression						
Leukopenia	0	2 (5)	0	0	0	2 (5)
Thrombocytopenia	0	0 (0)	1 (2.5)	0	0	1 (2.5)
Gastrointestinal reactions						
Nausea	1 (2.5)	1 (2.5)	0	0	0	2 (5)
Diarrhea	1 (2.5)	2 (5)	0	0	0	3 (7.5)
Fever	0	1 (2.5)	0	0	0	1 (2.5)
Pneumonitis	1 (2.5)	1 (2.5)	1 (2.5)	0	0	3 (7.5)
Laryngeal edema	0	1 (2.5)	0	0	0	1 (2.5)
Elevated transaminases	0	0	1 (2.5)	0	0	1 (2.5)
Limb numbness	0	1 (2.5)	0	0	0	1 (2.5)
Total	3 (7.5)	14 (35)	5 (12.5)	0	0	22 (55)

**TABLE 3:** Immune‐related adverse events.

	Grade 1	Grade 2	Grade 3	Grade 4	Grade 5	Total cases
	(*n*, %)	(*n*, %)	(*n*, %)	(*n*, %)	(*n*, %)	(*n*, %)
Skin rash	0	5 (12.5)	2 (5)	0	0	7 (17.5)
Diarrhea	1 (2.5)	2 (5)	0	0	0	3 (7.5)
Pneumonitis	1 (2.5)	1 (2.5)	1 (2.5)	0	0	3 (7.5)
Elevated transaminases	0	0	1 (2.5)	0	0	1 (2.5)
Total	2 (5)	8 (20)	4 (10)	0	0	14 (35)

### 3.3 Biomarker Studies

**FIGURE 4:**
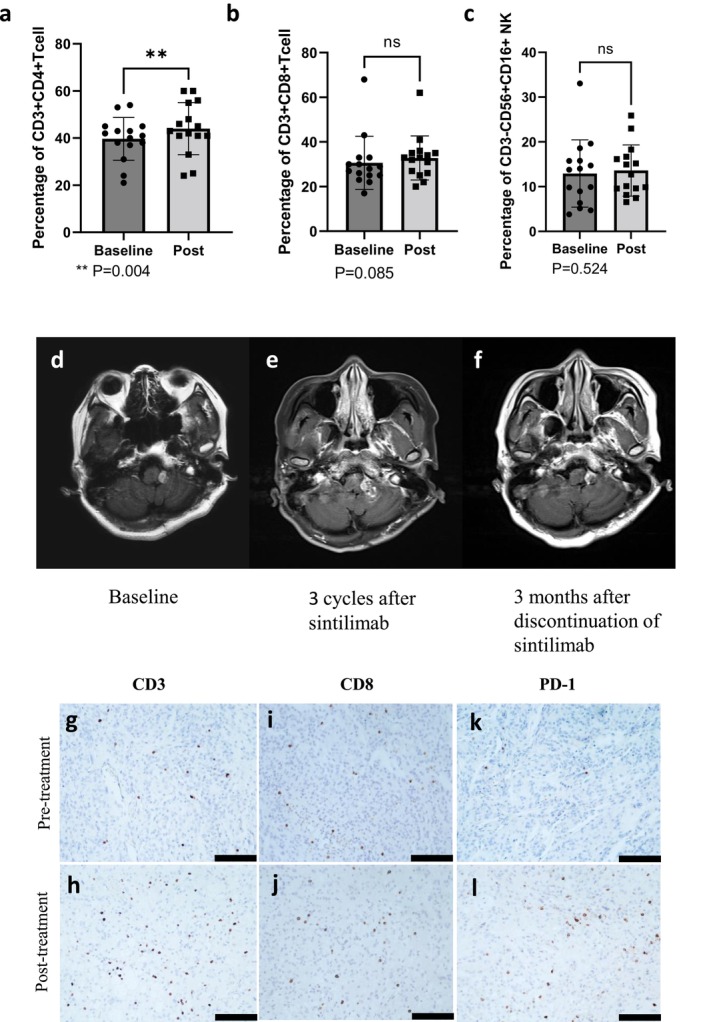
(a–c) Changes in lymphocyte subsets before and after treatment. (a) CD3 + CD4 + T cell. (b) CD3 + CD8 + T cell. (c) CD3‐CD56 + CD16 + NK. (d–f) Gadolinium‐enhanced MRI scan of patient 7. (d) Baseline. (e) Three cycles after sintilimab were conducted. (f) Three months after discontinuation of sintilimab. (g–l) Immunohistochemical images of tumor‐infiltrating lymphocytes pre‐ and post‐treatment of patient 24. Scale bars = 100 μm.

We examined peripheral blood lymphocyte subsets in 15 patients (3 grade 1, 7 grade 2, and 5 grade 3) pre‐ and post‐treatment after 2 cycles of treatment. Paired‐sample *t*‐test showed that in all grades, CD4+ T cell proportions were significantly elevated posttreatment (*p* = 0.004); the differences in CD8+ T‐cell and NK‐cell proportions were not statistically significant (Figure 4a–c).

We obtained tumor tissue and peripheral blood whole‐exome sequencing reports from 8 patients before enrollment. TMB and microsatellite instability (MSI) data were obtained from the sequencing reports and medical record instruments of these 8 patients, as shown in the table below (Table 4). The median TMB was 1.61 muts/Mb (range: 0.2–13.14 muts/Mb). Although no patients were observed to respond to treatment, we observed a case of a patient (no. 7) with a high TMB who had a pseudoprogression with PD‐1 inhibitor and maintained SD among subsequent treatments. This patient is a 30‐year‐old female diagnosed with meningioma in 2019, underwent surgical total resection without radiotherapy in January 2020, pathologically anaplastic meningioma (grade II–III), whole genome sequencing showed TMB = 13.14 muts/Mb, microsatellite stable, mutated genes: NOTCH2, PMS2, POLD1. In April 2021, due to symptomatic progression she was consulted and underwent an enhanced MRI showing a dorsolateral occupancy of the left medulla oblongata (Figure 4d), after which treatment with sintilimab was initiated. After 3 cycles of treatment, the tumor was found to be enlarged by enhanced MRI (Figure 4e), and the treatment was suspended without any significant change in symptoms. Subsequently, the patient received no glucocorticoids or other antitumor therapy and showed no progression of symptoms. Enhanced MRI was reviewed again 3 months after discontinuation of sintilimab (6 months from baseline) and showed no significant progression from baseline (Figure 4f), suggesting the possibility of pseudoprogression. Thereafter, sintilimab treatment was continued for 8 cycles without symptomatic progression, and local review of the enhanced MRI showed a 12% reduction in the area of the enhanced portion of the tumor from baseline.

**TABLE 4:** Summary of clinical characteristics and TMB in 8 patients.

Patient number	Grade	TMB	MSI	PFS	OS
		muts/Mb			
7	3	13.14	MSS	3	27
1	3	1.76	MSS	4	10
10	3	1.45	MSS	10	12+
20	2	5.7	MSS	21	47+
25	2	5.16	MSS	4	4
22	2	0.46	—	7	32+
33	1	1.0	MSS	6	46+
38	1	0.2	—	19	46+

Abbreviations: MSI, microsatellite instability; MSS, microsatellite stable; OS, overall survival; PFS, progression‐free survival; TMB, tumor mutation burden.

Based on our follow‐up, 5 patients underwent surgical resection after completing immunotherapy. We obtained paired pre‐ and post‐immunotherapy FFPE tumor blocks from 3 patients and performed immunohistochemical analysis of TILs (Figure S1). Figure 4g–l presents immunohistochemical images of TILs in patient 24 before and after sintilimab (provided by the patients themselves; informed consent was obtained). The 35‐year‐old male was diagnosed with a left frontal meningioma in 2018 and underwent gross total resection (GTR). Pathology confirmed “atypical meningioma (WHO Grade II).” In February 2020, the tumor recurred and he underwent a second GTR followed by radiotherapy. However, the tumor soon recurred in September 2020 (pretreatment). Later, he received 12 cycles of sintilimab at our hospital, with the best response being SD. Contrast‐enhanced MRI showed tumor progression in June 2021. Four months later he underwent surgical resection (posttreatment). The results demonstrated an increase in CD3+ and PD‐1+ T cell populations.

## 4 Discussion

In this study, we reported a clinical trial result of the PD‐1 inhibitor sintilimab in recurrent/progressive meningioma. The primary efficacy assessment metric set for this study was PFS‐6, based primarily on a meta‐analysis published by the RANO organization in 2014 [18]. Although the result of grade 2/3 was higher than the 26% benchmark established by RANO for systemic agents, it did not surpass the prespecified significance threshold of 51% defined by the statistical assumptions for our sample size.

The results of the phase 2 clinical trial of nivolumab for recurrent high‐grade meningiomas conducted by Bi et al. [19] showed a PFS‐6 of 42.4%, and the results of the phase 2 clinical trial of pembrolizumab for recurrent high‐grade meningiomas conducted by Brastianos et al. [20] showed a PFS‐6 of 48%. All of which were above the threshold of interest previously defined by the RANO group in 2014. However, recent evidence from a meta‐analysis conducted by Kotecha et al. [21], which incorporated the latest research findings, suggests that the predetermined benchmarks may have been excessively conservative. Their analysis established new thresholds of 67% for PFS‐6 and 54% for PFS‐12 in grade 1 tumors, with an adjusted PFS‐6 benchmark of 49% for grade 2/3 tumors. Compared with the new benchmark values, our study showed no improvement in PFS‐6 for either grade 1 or grade 2/3 meningiomas. Consistent findings were obtained both when applying the 2014 criteria‐defined endpoints and when conducting post hoc analyses using standards conducted in 2025, collectively demonstrating that sintilimab does not enhance PFS‐6 in patients with recurrent/progressive meningioma. Overall, our results did not demonstrate improvement over the benchmark values, and the enrolled populations exhibited significant heterogeneity. Additionally, the sample size was relatively small, and this study was a single‐arm trial lacking a control group. Furthermore, PFS‐6 as a primary endpoint has limitations in fully capturing the differences within the target population. Therefore, while this study provides valuable preliminary evidence regarding the potential efficacy of sintilimab in recurrent/progressive meningioma, the current findings are insufficient to establish PD‐1 inhibitors as a definitive treatment option for this disease.

Previous studies have indicated that biomarkers associated with the efficacy of ICIs can be broadly categorized into two groups: those related to tumor neoantigen burden, such as MSI or high TMB [22, 23]; and those indicative of a T‐cell‐inflamed tumor microenvironment, including the expression of PD‐L1 on tumor and immune cells infiltration [24, 25, 26, 27]. To identify biomarkers associated with the prognosis of PD‐1 inhibitor treatment in recurrent meningiomas, we collected data on TMB and MSI. Additionally, immunohistochemical analysis was performed to assess TILs in 3 patients at baseline and after treatment. We obtained TMB data from 8 patients (2 in grade 1, 3 in grade 2, and 3 in grade 3), with a median value of 1.61 muts/Mb (range: 0.2–13.14 muts/Mb). This is lower than the median TMB values previously reported in meningiomas, which were 4.2 muts/Mb (with grade 1 at 4.0 muts/MB, grade 2 at 4.4 muts/MB, and grade 3 at 6.5 muts/Mb) [11, 15, 28, 29]. Survival analysis revealed no significant correlation between TMB and PFS/OS following PD‐1 inhibitor treatment. In the phase 2 trial conducted by Bi et al. [19], a patient with multiply recurrent atypical meningioma (WHO grade 2) exhibited a TMB of 38.0 muts/MB, along with MSH2 deficiency. This patient was the only one in the study to achieve a best response of PR. We obtained matched pre‐ and post‐ treatment tumor samples from 3 patients, with 2 cases demonstrating an upward trend in PD‐1+ T cell expression following sintilimab treatment (patient 24 and 17). Although limited by small sample size, our preliminary observations suggest that PD‐1 inhibition may induce pathological responses in recurrent/progressive meningiomas.

Due to immune cell infiltration and the time required for an effective immune response, patients receiving immunotherapy may initially demonstrate tumor volume increase followed by subsequent radiographic stabilization or regression. This phenomenon of pseudoprogression has also been documented in other tumor types [30]. The RANO group developed Immunotherapy Response Assessment in Neuro‐Oncology (iRANO) criteria [17] which is also cited by RANO‐meningioma to solve that. When it comes to immunotherapy, iRANO suggests that for patients who meet the criteria for PD according to RANO within 6 months of immunotherapy, treatment may be continued if there is no significant clinical deterioration. It is recommended to perform additional imaging examinations 3 months after suspected progression. If progression is confirmed, the actual progression date should be traced back to the initial imaging progression date. Given the limited documentation of pseudoprogression in meningioma immunotherapy, we consistently applied the conventional RANO‐meningioma criteria during treatment (discontinuing sintilimab upon PD, even when occurring before completing 6 months of therapy), while maintaining close clinical surveillance and conducting post hoc analyses according to iRANO criteria. In our post hoc analysis of 19 patients (2 grade 1, 8 grade 2, and 9 grade 3) demonstrating radiographic progression within 6 months, only one case (5.3%) was confirmed as pseudoprogression (patient no. 7). The grade 2 meningioma patient with a relatively high TMB (13.14 muts/Mb) and the aforementioned patient (grade 2, TMB = 38.0 muts/Mb) exhibited similarities in their treatment outcomes. Both patients had high TMB levels, developed pseudoprogression after 3 cycles of PD‐1 inhibitor treatment, and subsequently showed radiographic improvement. These two cases highlight that, when evaluating PD‐1 inhibitor treatment for recurrent/progressive meningioma patients, who generally have a longer expected survival and high TMB, the use of the iRANO criteria may be more appropriate to avoid overlooking potential clinical benefits.

As our study commenced prior to the publication of WHO CNS 5 [31], meningioma grading was based solely on histopathology without integrated molecular profiling (H3K27me3, TERT promoter, CDKN2A/B status undetermined). This did not affect the grouping of high‐grade meningiomas in our study, but may have led to underestimation of grade 1 tumors, potentially influencing clinical trial outcomes. A precise molecular classification of meningiomas should be established in subsequent investigations.

## Author Contributions

Z.W., W.L., and F.C. were involved in conceptualization. Y.W., C.W., S.Y., C.Y., X.L., X.K., S.Y., W.X., and Y.L. participated in data collection and analysis. Y.W. wrote the article. All authors contributed to the article and approved the submitted version.

## Funding

This research was funded by The National Key R&D Program of China (No. 2021YFF0901404) and the Beijing Clinical Key Specialty Project (2‐1‐2‐038).

## Ethics Statement

This study was conducted in strict accordance with the ethical principles outlined in the Declaration of Helsinki and Good Clinical Practice guidelines. The study protocol received ethical approval from the Institutional Review Board of Beijing Tiantan Hospital, Capital Medical University.

## Consent

Written informed consent was obtained from all participating patients.

## Conflicts of Interest

The authors declare no conflicts of interest.

## Supporting information


**Figure S1:** Immunohistochemical images of tumor‐infiltrating lymphocytes pre‐ and posttreatment of patient 18, 17 and 24.

**Appendix S1:** Trial protocol.

## Data Availability

The original contributions presented in the study are included in the article. Further inquiries can be directed to the corresponding author.
