# Supplementary material for: Phase 2 Trial of PD‐1 Inhibitor Sintilimab in Recurrent/Progressive Meningioma
Source: CNS Neurosci Ther. 2025 Dec 8;31(12):e70659. doi: 10.1111/cns.70659 (PMC12683679; doi:10.1111/cns.70659)
Supplement: Supplementary file 1 — Figure S1: Immunohistochemical images of tumor‐infiltrating lymphocytes pre‐ and posttreatment of patient 18, 17 and 24. [file CNS-31-e70659-s001.pptx]

## Slide 1
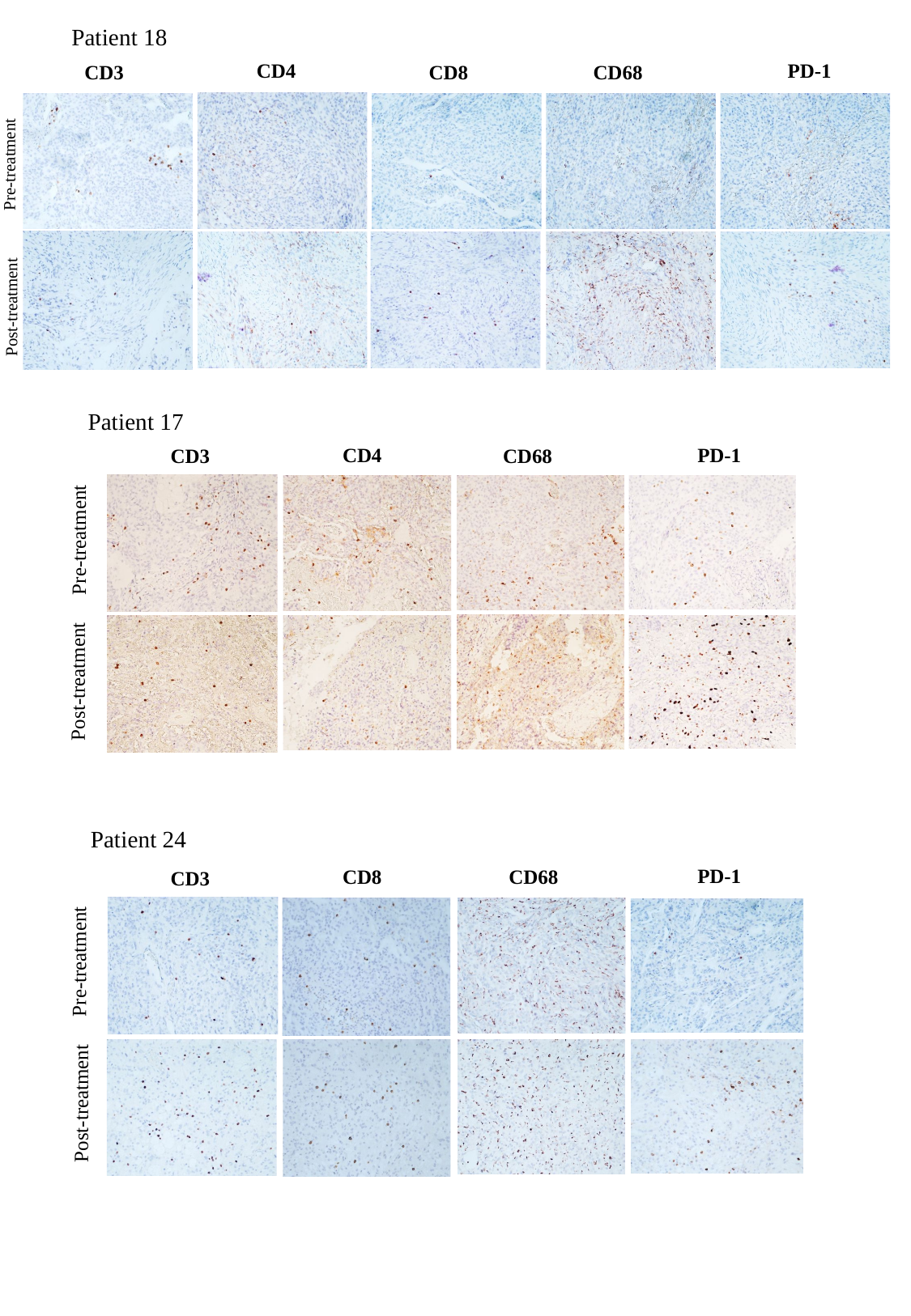

Patient 18
PD-1
CD4
CD3
CD68
CD8
Pre-treatment
Post-treatment
Patient 17
PD-1
CD4
CD68
CD3
Pre-treatment
Post-treatment
Patient 24
PD-1
CD68
CD8
CD3
Pre-treatment
Post-treatment
