# Supplementary material for: Phase 2 Trial of PD‐1 Inhibitor Sintilimab in Recurrent/Progressive Meningioma
Source: CNS Neurosci Ther. 2025 Dec 8;31(12):e70659. doi: 10.1111/cns.70659 (PMC12683679; doi:10.1111/cns.70659)
Supplement: Supplementary file 2 — Appendix S1: Trial protocol. [file CNS-31-e70659-s002.docx]

**Trial Protocol**

**Project summary**

Systemic therapeutic options for meningiomas remain limited. Here we conducted a phase 2 clinical trial (ClinicalTrials.gov: NCT 04728568) evaluating the efficacy and safety of programmed cell death receptor-1 (PD-1) inhibitor sintilimab in patients with recurrent/progressive meningiomas following standard surgery and/or radiotherapy. Patients with histologically confirmed meningioma who have received prior standard therapies (including surgery and/or radiotherapy) and are deemed by the investigator to be ineligible for further surgical intervention or radiotherapy at the current stage may be enrolled if they meet the following criteria for recurrence or progression: a) Recurrent disease: Clear tumor recurrence or progression after surgical resection. b) Progressive disease: Documented radiographic evidence of disease progression following the last medical intervention (surgery, radiotherapy, or drug therapy) in patients who have undergone prior surgery, or surgery combined with radiotherapy. According to Response Assessment in Neuro-Oncology for meningioma (RANO-meningioma) criteria, 6-month progression-free survival rate (PFS-6) was used as the primary endpoint. Secondary endpoints included 12-month progression-free survival rate (PFS-12), PFS, overall survival (OS) and safety. Peripheral lymphocyte subpopulations, tumor-infiltrating lymphocyte (TIL) densities, and tumor mutational burden (TMB) were evaluated as immunocorrelated biomarkers. For grade 2/3, based on the PFS-6 threshold (26%) established by the RANO working group, we hypothesized that sintilimab could increase PFS-6 to 51% (absolute improvement of 25%). The original design, with a one-sided α of 0.10 (exact α=0.089), required 25 patients to provide 90% statistical power, setting the success criterion as ≥10 cases (40%) achieving PFS-6.

**General information**

- Phase 2 Trial of PD-1 Inhibitor Sintilimab in Recurrent/Progressive Meningioma ClinicalTrials.gov: NCT 04728568 Date:2020-06-01 to 2025-06-01
- Sponsor: Feng Chen, Wenbin Li, Zhen Wu. Address: Beijing tiantan hospital,100070
- Zhen Wu, Department of Neurosurgery, Beijing Tiantan Hospital, Capital Medical University, Beijing,100070, China. E-mail: wuzhen1966@aliyun.com; Feng Chen, Department of Neuro-oncology, Cancer Center, Beijing Tiantan Hospital, Capital Medical University, Beijing,100070, China. E-mail: [chenfeng406@sina.com](mailto:chenfeng406@sina.com); Wenbin Li, Department of Neuro-oncology, Cancer Center, Beijing Tiantan Hospital, Capital Medical University; Beijing,100070, China. E-mail: liwenbin@ccmu.edu.cn.
- Yali Wang^a^, Can Wang^a,d^, Shuo Yin^a^, Chunna Yu^a,b^, Xiaojie Li^c^, Xun Kang^a^, Shoubo Yang^a^, Wenting Xie^a^, Yi Lina, Zhen Wu^c^, Feng Chen^a^, Wenbin Li^a^

a Department of Neuro-oncology, Cancer Center, Beijing Tiantan Hospital, Capital Medical University, Beijing, China.

b Beijing Luhe Hospital, Capital Medical University Beijing, China.

c Department of Neurosurgery, Beijing Tiantan Hospital, Capital Medical University, Beijing, China.

d Hepato-Pancreato-Biliary Center, School of Clinical Medicine, Beijing Tsinghua Changgung Hospital, Tsinghua University, Beijing, 102218, China.

**Rationale & background information**

Meningiomas represent the most common primary intracranial neoplasms, accounting for approximately one-third of all primary central nervous system tumors [1, 2]. According to the World Health Organization (WHO), the majority of meningiomas (80-95%) are classified into low grade (grade 1), while a smaller proportion are classified as high grade (grade 2: 5-15%, grade 3: 1-3%)[3-5]. These higher-grade meningiomas demonstrate aggressive clinical behavior, with recurrence rates reaching 30-40% for grade 2 and up to 80% for grade 3 tumors. The prognosis remains poor, with 5-year overall survival rates of only 60% and 30% for grade 2 and 3 meningiomas, respectively[6-9]. Despite multimodal treatment approaches including multiple surgical resections and radiotherapy, disease progression remains a significant challenge for many patients. Currently, systemic treatment options for meningiomas are limited, highlighting the urgent need for developing more effective therapeutic strategies.

In recent years, immune checkpoint inhibitors (ICIs) have achieved remarkable results in treating various solid tumors. Specifically, monoclonal antibodies targeting programmed cell death receptor-1 (PD-1) and programmed cell death-ligand 1 (PD-L1) have shown significant clinical benefits in malignancies such as lung cancer and melanoma ^[10]^ . However, current clinical guidelines do not include PD-(L)1 inhibitors as recommended treatment options for meningiomas, highlighting an important gap in therapeutic strategies for these intracranial tumors.

Growing evidence suggests that meningiomas possess an immunosuppressive tumor microenvironment. Notably, PD-L1 expression is significantly upregulated in both tumor cells and tumor-infiltrating immune cells, with expression levels correlating positively with histological grade^[10-13]^. This biological rationale is supported by promising responses to PD-1 inhibitors observed in case reports of recurrent meningiomas^[14, 15]^. These findings provide a strong mechanistic basis for investigating PD-1 blockade in recurrent meningiomas, particularly in high-grade cases. However, large-scale clinical studies evaluating PD-1 inhibitors in this population remain lacking, and both therapeutic efficacy and underlying mechanisms require systematic investigation.

**References (of literature cited in preceding sections)**

[1] Goldbrunner R, Minniti G, Preusser M, Jenkinson MD, Sallabanda K, Houdart E, von Deimling A, Stavrinou P, Lefranc F, Lund-Johansen M, Moyal EC-J, Brandsma D, Henriksson R, Soffietti R, Weller M (2016) EANO guidelines for the diagnosis and treatment of meningiomas. The Lancet Oncology 17: e383-e391.

[2] Zhu H, Bi WL, Aizer A, Lingyang Hua 1, Mi Tian 4, Jiaojiao Den 1, Hailiang Tang 1, Hong Chen 5, Wang Y (2019) Efficacy of adjuvant radiotherapy for atypical and anaplastic meningioma. Cancer Med 8: 13-20 <https://www.ncbi.nlm.nih.gov/pubmed/30680963>.

[3] Louis D, Perry A, Reifenberger G, von Deimling A, Figarella-Branger D, Cavenee WK, Ohgaki H, Wiestler OD, Kleihues P, Ellison DW (2016) The 2016 World Health Organization Classification of Tumors of the Central Nervous System: a summary. Acta Neuropathol 131: 803-20 <https://www.ncbi.nlm.nih.gov/pubmed/27157931>.

[4] Buerki RA, Horbinski CM, Kruser T, Horowitz PM, James CD, Lukas RV (2018) An overview of meningiomas. Future Oncol 14: 2161-2177.

[5] Wang C, Kaprealian TB, Suh JH, Kubicky CD, Ciporen JN, Chen Y, Jaboin JJ (2017) Overall survival benefit associated with adjuvant radiotherapy in WHO grade II meningioma. Neuro Oncol 19: 1263-1270 <https://www.ncbi.nlm.nih.gov/pubmed/28371851>.

[6] Goldbrunner R, Stavrinou P, Jenkinson MD, Sahm F, Mawrin C, Weber DC, Preusser M, Minniti G, Lund-Johansen M, Lefranc F, Houdart E, Sallabanda K, Le Rhun E, Nieuwenhuizen D, Tabatabai G, Soffietti R, Weller M (2021) EANO guideline on the diagnosis and management of meningiomas. Neuro Oncol 23: 1821-1834 <https://www.ncbi.nlm.nih.gov/pubmed/34181733>.

[7] Rogers L, Barani I, Chamberlain M, Kaley TJ, McDermott M, Raizer J, Schiff D, Weber DC, Wen PY, Vogelbaum MA (2015) Meningiomas: knowledge base, treatment outcomes, and uncertainties. A RANO review. J Neurosurg 122: 4-23.

[8] Zaher A, Abdelbari Mattar M, Zayed DH, Ellatif RA, Ashamallah SA (2013) Atypical meningioma: a study of prognostic factors. World Neurosurg 80: 549-53.

[9] Hanft S, Canoll P, Bruce JN (2010) A review of malignant meningiomas: diagnosis, characteristics, and treatment. J Neurooncol 99: 433-43.

[10] Li YD, Veliceasa D, Lamano JB, Lamano JB, Kaur G, Biyashev D, Horbinski CM, Kruser TJ, Bloch O (2019) Systemic and local immunosuppression in patients with high-grade meningiomas. Cancer Immunol Immunother 68: 999-1009 <https://www.ncbi.nlm.nih.gov/pubmed/31030234>.

[11] Du，Ziming, Abedalthagafi，Malak , Aizer A, Allison R McHenry 3 HHSMA, Sun HH, Bray MA, Viramontes O, Machaidze R, Brastianos PK, Reardon DA, Dunn IF, Freeman GJ, Ligon KL, Carpenter AE, Alexander BM, Agar NY, Rodig SJ, Brad (2015) Increased expression of the immune modulatory molecule PD-L1 (CD274) in anaplastic meningioma. Oncotarget. 6(7):4704-16.

[12] Han SJ, Reis G, Kohanbash G, Shrivastav S, Magill ST, Molinaro AM, McDermott MW, Theodosopoulos PV, Aghi MK, Berger MS, Butowski NA, Barani I, Phillips JJ, Perry A, Okada H (2016) Expression and prognostic impact of immune modulatory molecule PD-L1 in meningioma. J Neurooncol 130: 543-552 <https://www.ncbi.nlm.nih.gov/pubmed/27624915>.

[13] Karimi S, Mansouri S, Mamatjan Y, Liu J, Nassiri F, Suppiah S, Singh O, Aldape K, Zadeh G (2020) Programmed death ligand-1 (PD-L1) expression in meningioma; prognostic significance and its association with hypoxia and NFKB2 expression. Sci Rep 10: 14115 <https://www.ncbi.nlm.nih.gov/pubmed/32839486>.

[14] Gelerstein E, Berger A, Jonas-Kimchi T, Strauss I, Kanner AA, Blumenthal DT, Gottfried M, Margalit N, Ram Z, Shahar T (2017) Regression of intracranial meningioma following treatment with nivolumab: Case report and review of the literature. Clin Neurosci 37: 51-53 <https://www.ncbi.nlm.nih.gov/pubmed/28089420>.

[15] Dunn IF, Du Z, Touat M, Sisti MB, Wen PY, Umeton R, Dubuc AM, Ducar M, Canoll PD, Severson E, Elvin JA, Ramkissoon SH, Lin JR, Cabrera L, Acevedo B, Sorger PK, Ligon KL, Santagata S, Reardon DA (2018) Mismatch repair deficiency in high-grade meningioma: a rare but recurrent event associated with dramatic immune activation and clinical response to PD-1 blockade. JCO Precis Oncol <https://www.ncbi.nlm.nih.gov/pubmed/30801050>.

**Study goals and objectives**

- Primary Objective: To assess the 6-month progression-free survival rate (PFS-6) following treatment with sintilimab.
- Secondary Objectives: To evaluate the 12-month progression-free survival rate (PFS-12), PFS, OS, and safety profile.

**Study Design**

This is a prospective, single-arm, open-label, phase 2 clinical trial conducted at a single center.

**Patient Selection**

**Key Inclusion Criteria:**

- Age ≥18 years.
- Histologically confirmed meningioma with documented recurrence or progression after prior standard therapy (surgery and/or radiotherapy).
- Radiographically measurable disease as per RANO-meningioma criteria (longest diameter and perpendicular shortest diameter ≥10 mm).
- Karnofsky Performance Status (KPS) ≥60.
- Adequate bone marrow, hepatic, and renal function.

**Key Exclusion Criteria:**

- Dependence on corticosteroids (>5 mg/day dexamethasone or equivalent).
- Prior exposure to PD-1/PD-L1 inhibitors within 6 months.
- Radiographic progression within 12 weeks after radiotherapy or within 4 weeks after last systemic therapy.
- Primary spinal meningioma.
- Active autoimmune disease requiring systemic immunosuppression within 3 months.
- Known contraindications to PD-1 inhibitors.

**Study Treatment**

- Intervention: Sintilimab 200 mg administered by intravenous infusion every 3 weeks (Q3W).
- Treatment Duration: Treatment continues until disease progression (per RANO-meningioma criteria), occurrence of intolerable adverse events, voluntary withdrawal, or investigator decision.

**Efficacy Assessments:**

- Tumor response assessed by contrast-enhanced MRI at baseline and every 6–9 weeks thereafter, interpreted per RANO-meningioma criteria.

**Safety Assessments:**

- Adverse events monitored throughout the study and graded according to CTCAE v5.03.

**Immunological Monitoring:**

- Peripheral blood samples for immunophenotyping (lymphocyte subsets: CD3+CD4+, CD3+CD8+, NK cells) and laboratory analyses (hematology, metabolic panel) collected at:baseline (pre-treatment) and every 6-9 weeks throughout the treatment period.

**Statistical Analysis**

- Sample Size: The study initially planned to enroll 25 grade 2/3 meningioma patients to detect an increase in PFS-6 from 26% (historical control) to 51% (α=0.10, one-sided; power=90%).
- Statistical Methods: Time-to-event endpoints (PFS, OS) will be analyzed using Kaplan-Meier estimates and log-rank tests. Paired continuous variables will be compared using paired t-tests following normality confirmation (Shapiro-Wilk test). Analyses will be performed using SPSS 26.0 and GraphPad Prism 9.2.0.

**Expected outcomes of the study**

Sintilimab prolongs PFS-6 in patients with recurrent/progressive Meningioma

**Duration of the project**

Date:2020-06-01 to 2025-06-01

**Consent to participate**

Informed consent was obtained from all individual participants included in the study.

**Patient consent statement**

Written informed consent was obtained from all participating patients.

**Ethics**

This study was conducted in strict accordance with the ethical principles outlined in the Declaration of Helsinki and Good Clinical Practice guidelines. The study protocol received ethical approval from the Institutional Review Board of Beijing Tiantan Hospital, Capital Medical University.
